# You and I Both: Self-Compassion Reduces Self–Other Differences in Evaluation of Showing Vulnerability

**DOI:** 10.1177/01461672211031080

**Published:** 2021-07-22

**Authors:** Anna Bruk, Sabine G. Scholl, Herbert Bless

**Affiliations:** 1 University of Mannheim, Germany

**Keywords:** self-compassion, showing vulnerability, self–other differences, perspective-taking, beautiful mess effect

## Abstract

People tend to be overly critical of their own displays of vulnerability, whereas observers evaluate others’ showing of vulnerability rather positively (beautiful mess effect). We propose that self-compassion might buffer against such misperceptions of one’s own vulnerabilities. When confronted with challenging situations, self-compassionate people are kind to themselves, see adversity as inevitable, and face the difficulty of their circumstances without overexaggeration. Thus, we hypothesized reduced self–other differences in the evaluation of showing vulnerability in self-compassionate individuals. The hypothesis was addressed in four studies. The first two studies measured self-compassion either immediately (Study 1a) or substantially (Study 1b) before participants evaluated showing of vulnerability. Studies 2 and 3 tested the generalizability of the hypothesis across different situations as well as the discriminant validity of self-compassion’s role in the reduction of the beautiful mess effect. Implications for research and practice are discussed.

Imagine yourself in love with a friend and wondering whether to confess your feelings. Would you show your vulnerability by confessing your love? Other situations in which people often feel vulnerable include admitting a mistake or fear, asking for help, or sharing the results of one’s creativity (Brown, 2012). Out of fear, however, individuals often choose not to show their vulnerability (e.g., Lee, 1997). Yet, new research demonstrated that showing vulnerability is evaluated more positively by observers (i.e., people who witness others’ display of vulnerability) than actors (i.e., people who show their own vulnerability; Bruk et al., 2018).

Building on prior research that has documented these self–other differences in the evaluation of showing vulnerability, this research investigates the role of self-compassion in the emergence of this effect. Specifically, we propose that “treating oneself with kindness, recognizing one’s shared humanity, and being mindful when considering negative aspects of oneself” (Neff, 2011, p. 1) might act as a buffer against the negative evaluations of showing one’s vulnerability. Below, we address the core concepts of our research: showing vulnerability, self-compassion, and their hypothesized interrelation in turn.

## Showing Vulnerability

Showing vulnerability has been defined as an “authentic and intentional willingness to be open to uncertainty, risk, and emotional exposure in social situations in spite of fears” (Bruk et al., 2018, p. 192). Although showing vulnerability might be a difficult endeavor, its potential benefits include fulfilling close relationships (Sprecher et al., 2013), enhanced job performance and satisfaction (Brooks et al., 2015), better health (Cepeda-Benito & Short, 1998), or increased creativity and innovation (Brown, 2012). Yet, fears of rejection or negative evaluations (e.g., being perceived as weak or incompetent) often prohibit people from reaping the rewards associated with showing vulnerability (Lee, 1997; Rosenfeld, 1979). Therefore, it is crucial to verify whether such fears are justified.

Indeed, fears of negative evaluations after showing vulnerability may have some merit as, by definition, risk is present in vulnerable situations. Rather than investigating the validity of fear around showing vulnerability in absolute terms (“Is it risky to take a risk?”), a more fruitful approach, thus, may be to frame the question in relative terms (“Is it as risky as we think it is?”). Hence, the primary research avenue has been built on comparing the perception of actors with that of the observers. Following this route, several studies demonstrated actors’ overestimation of negative evaluations by observers in some specific vulnerable situations such as asking for help (Brooks et al., 2015) and disclosing personal information (Gromet & Pronin, 2009; Savitsky et al., 2001).

Going beyond isolated situations, further research has focused on demonstrating that the same pattern holds for all kinds of vulnerability display. In qualitative interviews, Brown (2012) observed that individuals tend to view showing vulnerability rather as a strength in others but perceive it rather as a weakness in themselves. Recent quantitative research has provided support for this claim. Bruk and colleagues (2018) demonstrated across a variety of vulnerable situations that showing vulnerability is evaluated more positively in others than in oneself. Reflecting both the potential positive and negative aspects of showing vulnerability, these self–other differences were labeled as the beautiful mess effect (BME).

Further studies have examined why the BME occurs (Bruk et al., 2018). One explanation for this evaluation mismatch has been found with the help of construal level theory (CLT; Trope & Liberman, 2010). According to CLT, the self is psychologically close and is associated with lower construal levels. As a consequence, the self is construed rather concretely and with a stronger focus on details as well as on more negative aspects, leading to relatively negative evaluations of one’s own vulnerability display. By contrast, others are psychologically more distant and, due to higher construal levels, are construed more abstractly, leading to a stronger focus on the bigger picture and more positive aspects. Such cognitive emphasis of the observers, in turn, results in rather positive evaluations of showing vulnerability (Bruk et al., 2018). Differences in affect, however, failed to explain the BME (Bruk et al., 2018), rendering it unlikely that other prominent theories in the literature on self–other differences such as “affective forecasting” (Wilson & Gilbert, 2005), “empathy gap” (Van Boven & Loewenstein, 2005), or “risk as feelings” (Loewenstein et al., 2001) are behind the perception mismatch in the evaluations of vulnerability displays (cf. Van Boven et al., 2012).

On one hand, the BME is in line with the findings in the broader literature on self–other differences in perception, for example, the tendency toward less risk aversion on behalf of others than for the self in relational domains (Stone et al., 2013) or people’s propensity to underestimate how much their conversation partners like them (Boothby et al., 2018). On the other hand, at first glance, the BME seems to contradict the literature showing that the self is often seen in a more positive light than others. For instance, for negative events, people are prone to dispositional attributions as observers while attributing their own behavior to situations (Ross, 1977). Such attribution errors tend to be of a self-serving nature (Mezulis et al., 2004), which can be partially traced back to the need to have and to convey a positive image (Shepperd et al., 2008). In addition, in part due to overestimating one’s control over outcomes, people exhibit a tendency toward unrealistic optimism around their own chances of success or challenges (Weinstein, 1980). Yet, in vulnerable situations, a reversed pattern emerges: a more negative view of oneself than others. Arguably, it is precisely the fact that people are invested in keeping a positive image of themselves and their capabilities that the self is so humbled in vulnerability situations—when the normally elevated self-image clashes with one’s limitations and lack of control. Consequently, rather than contradict the previous findings, the BME highlights their crucial boundary conditions.

Importantly, self–other discrepancies in how showing vulnerability is evaluated are potentially problematic as they may prevent individuals from showing their vulnerability—and in turn eliminate the potential benefits described above. Therefore, a crucial research question becomes: How can these differences in perception be overcome? In this article, we propose that self-compassion may hold a key answer.

## Self-Compassion

Self-compassion can be described as an extension of kindness and nonjudgmental attitude toward oneself during difficult times. This construct consists of three conceptually different but somewhat overlapping dimensions, and each dimension has two components: the presence and the lack of a specific characteristic. The first dimension of self-compassion includes self-kindness and reduced self-judgment. A kind response toward oneself in times of suffering entails treating oneself as one would treat a good friend—with care and understanding—as opposed to berating and criticizing oneself. The second dimension encompasses the components common humanity and reduced isolation. It refers to recognizing failures, mistakes, and suffering as an unavoidable part of life rather than feeling isolated with one’s difficulties. The final dimension consists of the components mindfulness and reduced overidentification. It entails clear awareness of the present moment as opposed to either ignoring one’s problems or overexaggerating the magnitude of one’s own failures and difficulties (Neff, 2011). Substantial research has documented these core conceptual considerations and their implications for a large variety of applications (see Barnard & Curry, 2011; for a critical discussion, see Muris et al., 2016; Neff, 2016).

## Self-Compassion and Showing Vulnerability

Given the support that self-compassion can provide in difficult times, we argue that a compassionate response to one’s own vulnerabilities can influence the perception of its display. After all, a rather negative evaluation of one’s vulnerability display has been shown to originate from actors’ focus on possible negative outcomes of making oneself vulnerable (Bruk et al., 2018), such as the risk of having to experience shame or other taxing emotions. These are precisely the moments when people need self-compassion the most. Through assuring actors that they can cope with any outcome of a vulnerable situation, self-compassion should help to deal with the cognitive emphasis on the drawbacks of showing vulnerability. Thus, self-compassion should help individuals to see their own vulnerability display in a more positive light, that is, closer to how they see it in others.

Arguably, the ability to alter the evaluation of showing vulnerability could be attributed to all three dimensions of self-compassion. Let us take admitting a mistake as an example of showing vulnerability. Individuals who treat themselves kindly and lack self-judgmental tendencies might be better able to forgive themselves for making a mistake instead of shaming themselves for it. Furthermore, when concentrating on feelings of common humanity rather than isolation, individuals might remind themselves that others also make mistakes. Finally, mindfulness and reduced overidentification might help to accept the mistake, lessening the need either to overexaggerate or to deny its significance. Such a compassionate response to one’s own vulnerability, in turn, might make it easier to make oneself vulnerable and to take responsibility for one’s mistake.

Several findings support this line of argument although this research does not directly pertain to showing vulnerability. For example, self-compassion has been reported to provide a buffer against anxiety in instances that threaten one’s ego (Leary et al., 2007). Relatedly, Albertson and colleagues (2015) showed that compared with a control group, self-compassion training led to a decrease in body shame and dissatisfaction as well as to an increase in body appreciation. These findings suggest that self-compassion can be helpful in dealing with another example of showing vulnerability: revealing physical imperfections. Moreover, the fact that self-compassion has been linked to less perfectionism (Brown, 2012; Williams et al., 2008) further supports the prediction that self-compassionate people should be emotionally equipped to deal with a vulnerable situation constructively. In addition, it was found that self-compassion can boost (state) authenticity by reducing the fear of negative evaluations and increasing optimism (Zhang et al., 2019).

Furthermore, highly self-compassionate people have been shown to rely less on avoidance and escape as coping strategies in stressful circumstances than less self-compassionate individuals (Allen & Leary, 2010), presumably due to reduced threat and higher (perceived) controllability of the stressful events (Chishima et al., 2018). In addition, self-compassion led people to experience less burnout (Durkin et al., 2016) or stress and shame after a stressful event (Ewert et al., 2018)—emotions that are typically present in vulnerable situations (Brown, 2012).

The above considerations focus on the actors’ perceptions. However, to assess the full impact of self-compassion on the evaluations of showing vulnerability, it is necessary to also consider the observers’ perspective as a reference point. Three ways are conceivable in which self-compassion might relate to the mismatch in evaluations of vulnerability displays. First, if self-compassion improves both the actors’ and observers’ evaluations in comparable magnitude—that is, no differences between individuals high versus low in self-compassion with respect to the BME—actors would still be more cautious about showing vulnerability than necessary. Second, if self-compassion improves the actors’ evaluations significantly above those of observers, then individuals might be inclined to be less cautious about showing vulnerability than warranted. Third, there is also a constructive option, in which self-compassion could shift actors’ evaluations closer to those of observers, that is, attenuate the self–other differences. Thus, it is necessary to look at the previous research on how self-compassion influences the way individuals relate to others. Such research, however, is not only scarce but also reveals mixed results.

On one hand, evidence suggests that self-compassion is positively linked to concerns about the well-being of others (Neff & Pommier, 2013) as well as to empathy for others (Fuochi et al., 2018). Yet, other studies detected either no relationship between self-compassion and compassion for others (Barnard & Curry, 2011; Beresford, 2016; Leary et al., 2007; López et al., 2018) or even an inverse correlation between the two constructs (Mills et al., 2018). Given the vast empirical support for the link between self-compassion and self-related outcomes as well as no clearly documented connection between self-compassion and other-focused variables, we hypothesize a positive relationship between self-compassion and the evaluations of the self and refrain from hypothesizing effects on the evaluations of others. Concurrently, as self-compassion fosters clearer rather than elevated self-view (Neff, 2011), we do not expect overly positive evaluations of one’s own vulnerability display in self-compassionate individuals. Therefore, in sum, we expect self-compassion to *attenuate* the BME.

## Overview of the Present Studies

The proposed interaction between self-compassion and role (oneself vs. others) was tested in four studies. In Study 1a, we measured self-compassion and then asked participants to imagine either themselves or another person in a vulnerable situation revolving around a love confession. In Study 1b, we measured self-compassion 1 month prior to subjecting participants to the scenario from Study 1a. In Studies 2 and 3, we tested both the generalizability of the moderation hypothesis across other vulnerable situations (Study 2: body image concerns; Study 3: admitting a mistake) and the discriminant validity of self-compassion’s role in the reduction of the BME with respect to self-esteem (Study 2) and neuroticism (Study 3).

As the effect size of the BME is relatively large (Bruk et al., 2018), for a moderator to have practical relevance, a priori power analyses were based on the goal of detecting a medium to large effect size. Assuming *f*² = .25, α = .05, and (1 – β) = .80, the G*Power analysis (Faul et al., 2009) suggested 48 participants for a regression model with one moderator and 65 participants for a model with two moderators. Exceeding these minimum requirements, we aimed for 60 participants for studies with one moderator (Studies 1a and 1b) and 100 participants for the studies with two simultaneous moderators (Studies 2 and 3; VanVoorhis & Morgan, 2007). We allowed, however, for small variations in this goal due to practical considerations. To increase power, we manipulated the factor role in a within-subjects design in a counterbalanced order. To ensure that the order of role manipulation did not interfere with the key findings, the analyses controlled for this variation. The data that support the findings of all four non-preregistered studies, the analyses code, the codebook, and the materials are openly available in OSF at https://osf.io/mvpsw/?view_only=06aa074594334f5aa3a7902d0e72c9cb.

## Study 1a

To test whether self-compassion moderates the BME, we measured self-compassion and then subjected participants to a vulnerable situation revolving around a love confession (Bruk et al., 2018) both from one’s own and from another person’s perspective. We hypothesized that the BME would be diminished for individuals who are high versus low on self-compassion.

### Method

In a computer-based experiment, 60 heterosexual or bisexual students of a German university (26 females; *M*_age_ = 22.2 years, *SD*_age_ *=* 3.5; 57 heterosexual; 1 bisexual; 2 did not specify sexual orientation) were asked to rate their level of self-compassion. Self-compassion was measured on a 7-point scale (1 = *strongly disagree*; 7 = *strongly agree*) with the German translation (Dyllick-Brenzinger, 2010) of the standard scale by Neff (2003; e.g., “I’m kind to myself when I’m experiencing suffering”).

Afterward, participants were randomly assigned to evaluate showing vulnerability in oneself and others in a counterbalanced order (gender was balanced across conditions). Participants read a text that asked them to imagine either themselves or another person of their gender (Stephanie/Stephan) in a vulnerable situation. In this situation, they were (or the other person was) in love with their best friend and were (was) considering confessing their love. Vividly describing the conflicting feelings, the scenario mentioned both the positive emotions associated with being in love and the fear of rejection. After long consideration, the protagonist (oneself vs. Stephanie/Stephan) confessed their feelings and was waiting for the reaction of the love interest (Bruk et al., 2018). After having read the scenario, participants indicated on a scale from 1 (*strongly disagree*) to 7 (*strongly agree*) whether saying “I love you” first was an act of showing vulnerability. Next, the dependent variable *evaluation of showing vulnerability* was measured with eight items: for example, “By showing my (her/his) vulnerability, I am (Stephanie/Stephan is) showing courage/weakness,” “Generally, when I (Stephan/Stephanie) show(s) my (his/her) vulnerability, other people find it repellent/desirable”; 1 = *strongly disagree;* 7 = *strongly agree* (Bruk et al., 2018; see full scale in Supplemental Material). Subsequently, participants were presented with the same material from the second perspective: Participants who had answered from their own perspective now received questions from Stephanie’s/Stephan’s perspective and those who had previously evaluated Stephanie’s/Stephan’s showing of vulnerability now received the material from their own perspective. Finally, participants were debriefed, thanked, and remunerated.^1^

### Results and Discussion

**Figure 1.**
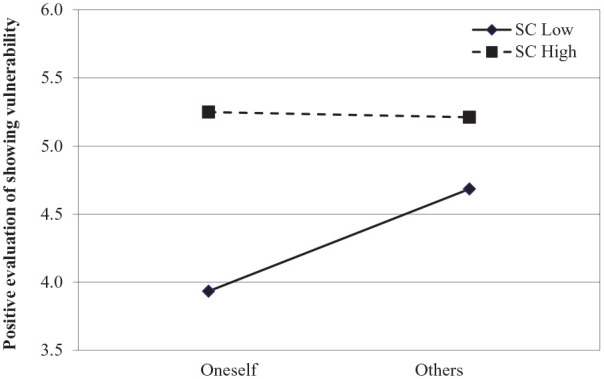
Positive evaluation of showing vulnerability as a function of role (oneself vs. others) and self-compassion from Study 1a. Lines are regression slopes from simple slopes analysis (low = 1 *SD* below the mean, high = 1 *SD* above the mean).

The items measuring the evaluation of showing vulnerability were averaged separately for *oneself* and *others.* Similarly, the self-compassion items were averaged and centered. To ensure that the observed effects cannot be attributed to differences in vulnerability perception between *oneself* and *others*, the difference between these perceptions was calculated and included as a control variable (for additional information, see Note 2; for descriptive statistics, see Table 1). A mixed factorial analysis of variance (ANOVA) with role as a within factor, order as a between factor, self-compassion as a continuous moderator, and differences in vulnerability perception as a control variable revealed a significant main effect of role. Replicating previous findings (Bruk et al., 2018), participants who imagined another person confessing their love evaluated showing vulnerability more positively than those who imagined themselves in the same situation (*M*_others_ = 4.95, *SD*_others_ = 1.04 vs. *M*_oneself_ = 4.59, *SD*_oneself_ = 1.16); *F*(1, 55) = 6.81, *p =* .01, $\eta_{p}^{2}=.11$ , 95% confidence interval (CI) = [.20, .54]. Furthermore, self-compassion was associated with a more positive evaluation of showing vulnerability; *F*(1, 55) = 19.05, *p <* .001, $\eta_{p}^{2}=.26$ . Most importantly, we found evidence for the hypothesized role by self-compassion interaction; *F*(1, 55) = 12.94, *p =* .001, $\eta_{p}^{2}=.19$ . Specifically, simple slopes analysis with Model 2 of Mediation and Moderation for Repeated Measures (MEMORE; Montoya, 2018) showed that when participants were low on self-compassion, showing vulnerability was evaluated more positively in others than oneself; β = .75, *t* = 5.63, *p* < .001, 95% CI = [.48, 1.02]. Conversely, for highly self-compassionate participants, the self–other differences were attenuated (|*t|* < 1; see Figure 1). In addition, the observed moderation of the BME through self-compassion occurred through the influence of self-compassion on the perception of one’s own vulnerability: β = .71, *t* = 5.27, *p* < .001, 95% CI = [.44, .98], whereas the evaluations of others’ vulnerability display improved through self-compassion only marginally: β = .28, *t* = 1.99, *p* =.051, 95% CI = [−.002, .57].

**Table 1.** Means, Standard Deviations, Internal Consistencies, and Intercorrelations Among Constructs. (Study 1a).

Construct		*M*	*SD*	α	1	2	3	4
1	Evaluation of SV (self)	4.59	1.16	.83	1.00			
2	Evaluation of SV (others)	4.95	1.04	.82	.73**	1.00		
3	Perception of vulnerability (self)	4.27	2.11	—	−.43**	−.09	1.00	
4	Perception of vulnerability (others)	3.77	1.95	—	−.40**	−.29*	.78**	1.00
5	Self-compassion	3.76	.93	.90	.57**	.25	−.33**	−.23

*Note*. SV = showing vulnerability.

** *p* < .01. *p < .05.

The present results provide first evidence that self-compassion moderates the BME: The self–other differences in the evaluation of showing vulnerability were reduced in self-compassionate individuals. Furthermore, this moderation effect occurred through an improvement in actors’ evaluations, while the observers’ evaluations were only marginally affected by self-compassion. In addition to demonstrating the moderating role of self-compassion, the findings provide a straightforward replication of the BME as, overall, participants evaluated showing vulnerability more positively in others than oneself (Bruk et al., 2018).^2–4^

## Study 1b

In Study 1a, we measured self-compassion right before participants evaluated showing vulnerability. One may argue that the observed moderation effect crucially depends on making self-compassion highly accessible prior to the evaluation of showing vulnerability. If so, the buffering effect of self-compassion would be restricted to rather few situations—and thus would have limited practical implications. To eliminate this concern, in Study 1b, we subjected our hypothesis to a more conservative test. We set out to replicate Study 1a with one important change: Self-compassion was measured 1 month before the main study, that is, substantially prior to the role manipulation. We predicted that the BME would still decrease for individuals who are high versus low in self-compassion.

### Method

At the beginning of the semester, students of a German university were invited to participate in a two-part study via email. The first part of the study consisted of an online questionnaire that assessed different traits and attitudes^5^ in a randomized order including the trait self-compassion (Dyllick-Brenzinger, 2010; Neff, 2003). This questionnaire was completed by 110 participants.

One month later, these participants were invited to the laboratory for the second part of the study. Eighty-five individuals participated in the laboratory part that included three independent online experiments conducted one after another on unrelated topics. The present study was the first of these three experiments. To match the responses of participants from the non-laboratory and the laboratory parts of the study while preserving anonymity, we asked participants to generate a unique code in both study parts. Using this code, 84 out of the 85 participants could be matched with their responses from the non-laboratory study part that measured self-compassion. Furthermore, because the scenario was applicable only for participants attracted to the opposite sex, we excluded the responses provided by two homosexual participants. Thus, the final sample included responses from 82 participants. Participation in both parts of the present study (as well as in the two unrelated studies conducted afterward) was rewarded either with EUR 12 or course credit.

The laboratory part was a computer-based study in which heterosexual or bisexual students of a German university (64 females; *M*_age_ = 20.4 years, *SD*_age_ *=* 3.3; 77 heterosexual; 1 bisexual; 4 did not specify sexual orientation) were randomly assigned to evaluate showing vulnerability in oneself and in others in a counterbalanced order. The manipulation of the factor role was identical to Study 1a.

### Results and Discussion

**Figure 2.**
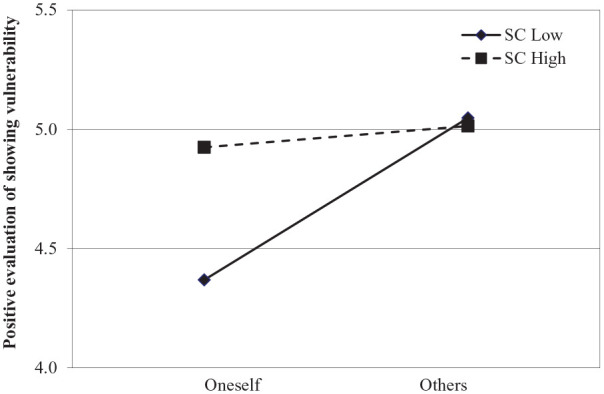
Positive evaluation of showing vulnerability as a function of role (oneself vs. others) and self-compassion from Study 1b. Lines are regression slopes from simple slopes analysis (low = 1 *SD* below the mean, high = 1 *SD* above the mean).

Again, we averaged the items measuring the evaluation of showing vulnerability separately for *oneself* and *others*, as well as averaged and centered the self-compassion items (for descriptive statistics, see Table 2). A mixed factorial ANOVA with role as a within factor, order as a between factor, self-compassion as a moderator, and differences in vulnerability perception as a control variable revealed a significant main effect of role: In line with Study 1a and previous findings (Bruk et al., 2018), when participants depicted another person confessing love, they evaluated showing vulnerability more positively than when they imagined themselves in the same situation (*M*_others_ = 5.03, *SD*_others_ = 0.80 vs. *M*_oneself_ = 4.64, *SD*_oneself_ = 1.02); *F*(1, 77) = 18.15, *p <* .001, $\eta_{p}^{2}=.119$ , 95% CI = [.20, .54]. In contrast to Study 1a, self-compassion had no overall effect on the evaluation of showing vulnerability; *F*(1, 77) = 1.67, *p =* .20, $\eta_{p}^{2}=.02$ . Most importantly, we replicated the role by self-compassion interaction; *F*(1, 77) = 10.55, *p =* .002, $\eta_{p}^{2}=.12$ . Participants low on self-compassion evaluated showing vulnerability more positively in others than oneself; β = .68, *t* = 5.60, *p* < .001, 95% CI = [.44, .92]. By contrast, in highly self-compassionate participants, the BME was attenuated (*t* < 1; see Figure 2). Finally, the observed moderation of the BME through self-compassion occurred due to the influence of self-compassion on the actors’ evaluations; β = .37, *t* = 2.53, *p* =.01, 95% CI = [.08, .66]. The effect of self-compassion on observers’ evaluations was nonsignificant; |*t|* < 1.

**Table 2.** Means, Standard Deviations, Internal Consistencies, and Intercorrelations Among Constructs. (Study 1b).

Construct		*M*	*SD*	α	1	2	3	4
1	Evaluation of SV (self)	4.65	1.02	.80	1.00			
2	Evaluation of SV (others)	5.03	0.80	.73	.61**	1.00		
3	Perception of vulnerability (self)	4.91	1.93	—	−.21	−.11	1.00	
4	Perception of vulnerability (others)	4.41	1.99	—	−.22*	−.10	.77**	1.00
5	Self-compassion	3.68	0.75	.89	.27*	−.02	−.15	−.14

*Note*. SV = showing vulnerability.

** *p* < .01. *p < .05.

Irrespective of whether self-compassion was measured right before (Study 1a) or separate from the induction of role (Study 1b), we found support for our moderation hypothesis: The BME was attenuated in self-compassionate individuals. Furthermore, this effect was driven by the improvement in the evaluations of one’s own vulnerability.

On one hand, the results of the first two studies provide consistent support for the outlined moderation hypothesis. On the other hand, however, the empirical tests were restricted to one specific vulnerable situation: a love confession. To allow for more generalization, it is necessary to incorporate other vulnerable situations as well. Moreover, given that self-compassion may co-vary with other variables, the present findings do not rule out that it is not self-compassion but other correlated traits that are the key drivers of the obtained findings. The subsequent two studies were designed to address these concerns by (a) employing different situational settings and (b) investigating the role of two traits that, based on prior research, seem the most likely candidates for alternative explanations—self-esteem and neuroticism.

## Study 2

One may argue that the observed moderation pattern is due to self-esteem—the extent to which the self is evaluated as competent in important life domains (James, 1890/1983)—rather than to self-compassion. After all, both traits represent positive attitudes toward oneself. Thus, it seems plausible that people who are high (low) in self-compassion would also have a relatively high (low) sense of self-worth given that they treat themselves kindly (harshly; Neff, 2011). Correspondingly, studies repeatedly found a substantial positive relationship between self-compassion and self-esteem (correlation ranging from .57 to .59; Leary et al., 2007; Neff, 2003, 2011).

Despite this correlation, however, previous research has also shown that the two traits have unique effects. For instance, self-compassion is positively correlated with happiness, positive affect, and optimism (Neff & Vonk, 2009) and is negatively correlated with anxiety and depression (Neff, 2003), even when controlling for self-esteem. Furthermore, Leary and colleagues (2007) observed that, compared to individuals with high self-esteem, participants high in self-compassion assumed more personal responsibility for their actions while also being kinder to themselves. In addition, Seekis and colleagues (2017) reported that participants who were presented with a negative body image scenario showed higher body appreciation after a self-compassion induction than after a self-esteem promoting exercise.

Neff (2011) argues that the reason why these two sources of positive self-regard have unique effects is because self-compassion has a supportive function precisely when self-esteem fails. It is in the moments when people stumble and face their imperfections—in other words, when they feel vulnerable—that they need self-compassion the most. Given that self-esteem tends to be contingent on the quality of one’s own performance relative to the performance of others, it can ﬂuctuate with recent instances of success or failure and can result in rumination about the implications of the setbacks (Neff, 2011). Therefore, self-esteem might not be able to offer protection in vulnerable situations that can threaten a positive self-view. Self-compassion, however, is not necessarily a form of positive self-evaluation but rather a positive way of treating oneself independent of performance—one’s own or others’. Consequently, it can be more stable than self-esteem (Neff, 2011). Thus, although self-esteem has been shown to play a role in reducing self–other differences in risky situations (Wray & Stone, 2005), on the basis of the above considerations, we postulate that self-compassion moderates the BME beyond the possible effects of self-esteem.

Besides disentangling the influence of self-compassion and self-esteem, Study 2 was designed to test the generalizability of the moderating effect observed in Studies 1a and 1b. As both self-compassion and self-esteem have been linked to reduced body image concerns (Albertson et al., 2015; O’Dea & Abraham, 2000), we chose “revealing physical imperfections” as an alternative vulnerable situation. We predicted the BME to be weaker for self-compassionate people in this situation as well.

### Method

After excluding the answers of three homosexual participants (see above), the final sample included 97 heterosexual or bisexual students of a German university (55 females; *M*_age_ = 21.8 years, *SD*_age_ *=* 2.6; 91 heterosexual; 6 did not specify sexual orientation).

The procedure was similar to Study 1a with the following changes: First, the vulnerable situation revolved around revealing physical imperfections to a love interest (Bruk et al., 2018). Second, self-compassion and self-esteem were assessed after participants had read the scenarios from different perspectives and had filled out the questionnaire for the dependent variables—this way, the trait measurements could not interfere with the manipulation of role. We measured self-esteem (Rosenberg, 1965; translated by Ferring & Filipp, 1996) on a scale from 1 (*strongly disagree*) to 7 (*strongly agree*). Finally, participants were debriefed, thanked, and remunerated with €1 and a chocolate bar or course credit.

### Results and Discussion

The items measuring the evaluation of showing vulnerability were averaged separately for *oneself* and *others*. Furthermore, the items assessing self-compassion and self-esteem were averaged, centered, and included as continuous moderators in a mixed factorial ANOVA with role as a within factor, order as a between factor, and differences in vulnerability perception as a control variable (for descriptive statistics, see Table 3). Replicating the pattern of Studies 1a and 1b, participants who imagined another person revealing physical imperfections evaluated showing vulnerability more positively than when they imagined themselves in the same situation (*M*_others_ = 4.65, *SD*_others_ = 0.98 vs. *M*_oneself_ = 4.23, *SD*_oneself_ = 0.98); *F*(1, 88) = 28.33, *p <* .001, $\eta_{p}^{2}=.24$ , 95% CI = [.32, .62]. The main effects of self-compassion and self-esteem as well as their interaction were nonsignificant; *F*s < 1.62 and *p*s > .20.

**Table 3.** Means, Standard Deviations, Internal Consistencies, and Intercorrelations Among Constructs. (Study 2).

Construct		*M*	*SD*	α	1	2	3	4	5
1	Evaluation of SV (self)	4.23	.98	.75	1.00				
2	Evaluation of SV (others)	4.65	.98	.79	.68**	1.00			
3	Perception of Vulnerability (self)	3.76	1.85	—	−.18	.13	1.00		
4	Perception of Vulnerability (others)	3.41	1.85	—	−.09	−.17	.46**	1.00	
5	Self-compassion	3.91	.82	.88	.29**	−.03	−.37**	−.09	1.00
6	Self-esteem	5.32	1.05	.88	.22*	.00	−.21*	−.02	.55**

*Note*. SV = showing vulnerability.

** *p* < .01. *p < .05.

**Figure 3.**
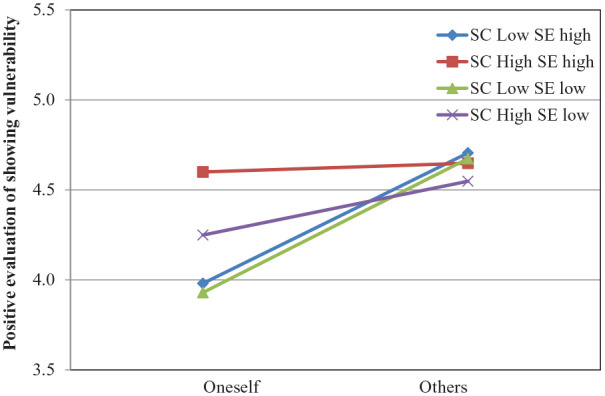
Positive evaluation of showing vulnerability as a function of role (oneself vs. others), self-compassion, and self-esteem from Study 2. Lines are regression slopes from simple slopes analysis (low = 1 *SD* below the mean, high = 1 *SD* above the mean).

Most importantly, the analyses revealed the hypothesized pattern: The role by self-compassion interaction was significant; *F*(1, 88) = 6.21, *p =* .02, $\eta_{p}^{2}=.07$ , whereas the role by self-esteem interaction was nonsignificant (*F* < 1). The three-way interaction between role, self-compassion, and self-esteem was nonsignificant as well; *F*(1, 88) = 1.87, *p =* .18, $\eta_{p}^{2}=.02$ . To gain more insight into the role by self-compassion interaction, we decomposed it using Model 3 of MEMORE (Montoya, 2018) and tested the effect of self-compassion on the evaluation of showing vulnerability at low and high levels of the trait (mean ±1 *SD*), both for low and high levels of self-esteem (mean ±1 *SD*). The analyses revealed that, independent of self-esteem, participants low on self-compassion evaluated showing vulnerability more positively in others than oneself (high self-esteem: β = .72, *t* = 3.84, *p* < .001, 95% CI = [.35, 1.10]; low self-esteem: β = .74, *t* = 6.58, *p* < .001, 95% CI = [.52, .97]). By contrast, independent of self-esteem, for highly self-compassionate participants, the self–other differences were attenuated (high self-esteem: *t* < 1; low self-esteem: *β* = .30, *t* = 1.45, *p =* .15, 95% CI = [−.11, .71]; see Figure 3). Moreover, this attenuation of self–other differences occurred due to self-compassion’s improvement of actors’ evaluations: β = .29, *t* = 2.03, *p* < .05, 95% CI = [.01, .57]. The effect of self-compassion on observers’ evaluations was nonsignificant. Finally, self-esteem did not significantly impact either the actors’ or the observers’ evaluations; all |*t*|s < 1.

Replicating the findings of Study 1a and 1b, the obtained results demonstrate again that self-compassion attenuates the BME and that this effect occurs due to an improvement of the evaluations of one’s own vulnerability display. Importantly, this moderation effect was observed independent of participants’ level of self-esteem. In contrast to self-compassion, self-esteem did not significantly influence the evaluation of showing vulnerability—neither when the evaluation pertained to one’s own nor to others’ vulnerability. This pattern suggests that self-compassion’s moderation of the BME does not result from individuals’ self-esteem (and from the overlap of these two traits). In addition to demonstrating the unique contribution of self-compassion, the present findings further indicate the generalizability of the moderation effect. Switching the scenarios from “love confession” to “revealing physical imperfections” did not change the observed pattern.

## Study 3

Neuroticism—a “predisposition to experience negative affect” (Gunthert et al., 1999, p. 1087)—constitutes another trait that could potentially account for the role of self-compassion in the reduction of the BME due to a high negative correlation between the two traits (*r* = −.65; Neff et al., 2007). Given this high correlation, Pfattheicher and colleagues (2017) have questioned whether having self-compassion is substantially different from lacking neuroticism and presented data showing that self-compassion cannot explain considerable incremental variance in life satisfaction beyond neuroticism. Other studies, however, demonstrated the incremental predictive validity of self-compassion. For instance, even when controlling for neuroticism, self-compassion predicted higher well-being (Neff et al., 2007, 2018), lower negative affect, depression, and anxiety (Stutts et al., 2018), as well as less perceived stress and shame (Ewert et al., 2018).

Conceptual differences between the two constructs might explain their unique effects on various outcomes. Neff and colleagues (2018) argue that neuroticism items tap into different dimensions of negative affect, such as anxiety (e.g., “I often feel tense and jittery”), measuring general, habitual negative mood-states without referencing the individual’s response to suffering. By contrast, low self-compassion indicates uncompassionate ways of relating to oneself in difficult times (e.g., “When times are really difficult, I tend to be tough on myself”). We argue that it is precisely the ability of self-compassion to put things in perspective and to respond with self-care when experiencing negative affect or when confronted with one’s own neurotic tendencies in vulnerable situations that allows individuals high on this trait to deal with vulnerable situations constructively. In line with this reasoning, Ewert and colleagues (2018) found that self-compassion can buffer the effect of neuroticism on the use of denial when coping with stressful situations. Thus, individuals who can employ self-soothing techniques may be more open-minded and open-hearted toward their own vulnerabilities beyond what can be explained by a low propensity to experience negative thoughts and feelings. Based on these considerations, we hypothesize that self-compassion moderates the emergence of the BME even when accounting for individuals’ level of neuroticism.

In addition to disentangling the effects of self-compassion and neuroticism, the present study further examined the generalizability of the moderation hypothesis in yet another vulnerable situation: admitting a mistake. If neuroticism plays a role in the evaluation of showing vulnerability, its effect should be especially pronounced in a situation that directly taps into neurotic tendencies, such as being confronted with one’s own mistakes. However, assuming that self-compassion makes people more likely to take personal responsibility (Leary et al., 2007), we predicted that self-compassion moderates the emergence of the BME in this new situation as well.

### Method

A total of 101 students of a German university (48 female; *M*_age_ = 22.1 years, *SD*_age_ *=* 3.8) participated in a computer-based study. The procedure was the same as in Study 2, except for the following changes. First, we changed the scenario: The vulnerable situation now revolved around confessing a mistake to one’s boss (Bruk et al., 2018). Second, after assessing self-compassion, we measured neuroticism with the 12 relevant Neuroticism-Extraversion-Openness Five-Factor Inventory *(*NEO-FFI) items (Costa & McCrea, 1992; translated by Borkenau & Ostendorf, 2008) on a scale from 1 to 5 (1 = *strongly disagree*; 5 = *strongly agree*).

### Results and Discussion

The items measuring the evaluation of showing vulnerability were averaged separately for *oneself* and *others*. Furthermore, the items assessing self-compassion and neuroticism were averaged, centered, and included as continuous moderators in a mixed factorial ANOVA with role as a within factor, order as a between factor, and differences in vulnerability perception as a control variable (for descriptive statistics, see Table 4). As in previous studies (Bruk et al., 2018), participants who imagined another person admitting a mistake evaluated showing vulnerability more positively than those who imagined themselves in the same situation (*M*_others_ = 4.92, *SD*_others_ = 0.81 vs. *M*_oneself_ = 4.65, *SD*_oneself_ = 0.90); *F*(1, 92) = 13.99, *p <* .001, $\eta_{p}^{2}=.13$ , 95% CI = [.13, .40]. In addition, there was a trend toward more positive evaluations of showing vulnerability on behalf of self-compassionate individuals: *F*(1, 92) = 3.42, *p =* .07, $\eta_{p}^{2}=.04$ . The main effect of neuroticism as well as its interaction with self-compassion were, however, nonsignificant; all *F*s < 1.

**Table 4.** Means, Standard Deviations, Internal Consistencies, and Intercorrelations Among Constructs. (Study 3).

Construct		*M*	*SD*	α	1	2	3	4	5
1	Evaluation of SV (self)	4.65	0.90	.76	1.00				
2	Evaluation of SV (others)	4.92	0.81	.73	.71**	1.00			
3	Perception of vulnerability (self)	3.82	1.85	—	−.26**	−.16	1.00		
4	Perception of vulnerability (others)	3.58	1.86	—	−.25*	−.32**	.71**	1.00	
5	Self-compassion	3.71	0.86	.91	.37**	.18	−.29**	−.22*	1.00
6	Neuroticism	2.84	0.71	.86	−.27**	−.10	.26**	.23*	−.72**

*Note*. SV = showing vulnerability.

** *p* < .01. *p < .05.

**Figure 4.**
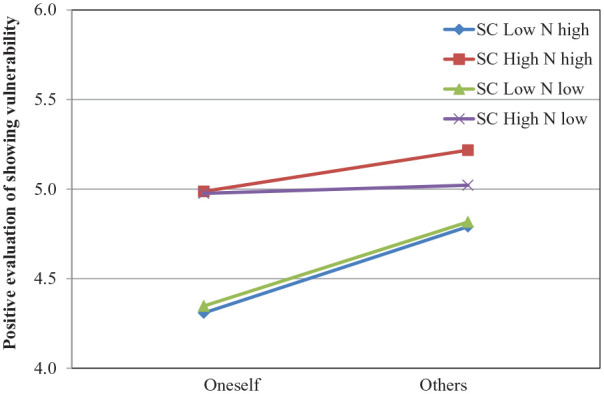
Positive evaluation of showing vulnerability as a function of role (oneself vs. others), self-compassion, and neuroticism from Study 3. Lines are regression slopes from simple slopes analysis (low = 1 *SD* below the mean, high = 1 *SD* above the mean).

Most important was the emergence of the predicted role by self-compassion interaction: *F*(1, 92) = 9.33, *p =* .003, $\eta_{p}^{2}=.10$ . By contrast, the two-way interaction between role and neuroticism was nonsignificant (*F* < 1). The three-way interaction between role, self-compassion, and neuroticism was nonsignificant as well; *F*(1, 92) = 1.17, *p =* .28, $\eta_{p}^{2}=.01$ . We used Model 3 of MEMORE (Montoya, 2018) to test the effect of self-compassion on the evaluation of showing vulnerability at low and high levels of the trait (the mean ±1 *SD*), both for low and high levels of neuroticism (the mean ±1 *SD*). The analyses revealed that, independent of neuroticism, participants who were low on self-compassion evaluated showing vulnerability more positively in others than oneself (high neuroticism: β = .48, *t* = 5.13, *p* < .001, 95% CI = [.30, .67]; low neuroticism: β = .47, *t* = 2.08, *p* = .04, 95% CI = [.02, .92]). By contrast, in highly self-compassionate individuals, the self–other differences were attenuated (high neuroticism: β = .23, *t* = 1.19, *p* = .24, 95% CI = [−.15, .61]; low neuroticism: *t* < 1); see Figure 4. Moreover, this attenuation of self–other differences occurred due to self-compassion’s improvement of actors’ evaluations: β = .38, *t* = 2.59, *p* = .01, 95% CI = [.08, .67]. The effect of self-compassion on observers’ evaluations was nonsignificant: β = .18, *t* = 1.32, *p* = .19, 95% CI = [−.09, .46]. Finally, neuroticism did not significantly impact either self-evaluations or those of others; all |*t|*s < 1.

Providing additional evidence for our moderation hypothesis, the present study replicated the previous findings that the BME is diminished in self-compassionate individuals and that this effect occurs through the improvements in the evaluations of one’s own vulnerability display. Most importantly, the moderating effect of self-compassion emerged independent of participants’ level of neuroticism. By showing that the observed moderation through self-compassion cannot be attributed to neuroticism, these results further demonstrate the discriminant validity of self-compassion as a moderator of the BME. In addition, the present results provide further evidence for the generalizability of the moderation effect by testing it in yet another vulnerable situation: admitting a mistake.^6^

## General Discussion

Results obtained in four studies consistently demonstrate that self-compassion moderates the differences between actors and observers in the evaluation of showing vulnerability. Specifically, individuals low in self-compassion evaluated showing vulnerability more positively in others than in oneself. This actor–observer difference reflects the BME that has been documented in prior research (Bruk et al., 2018), providing supporting evidence on the stability of this effect. Most importantly, however, the BME was less pronounced in individuals high in self-compassion. This pattern emerged consistently across three different settings: confessing love (Studies 1a and 1b), revealing physical imperfections (Study 2), and admitting a mistake (Study 3). Moreover, the findings were unaffected by whether self-compassion was measured prior to (Study 1a), after (Studies 2 and 3), or separate (Study 1b) from the evaluation of vulnerability.

In all four studies, in contrast to low levels of self-compassion, high levels of self-compassion were associated with reduced self–other differences. Given that actor-observer differences in the evaluation of showing vulnerability originate from the actors’ focus on possible negative outcomes of making oneself vulnerable (Bruk et al., 2018), the present findings suggest that self-compassion may serve as a buffer against anxiety (see Neff et al., 2007) and as protection against stress and shame (see Ewert et al., 2018) in vulnerable situations—emotions that may be present in all vulnerable situations. Thus, the obtained findings converge with the existing research on the consequences of self-compassion, indicating that self-compassionate people are more emotionally prepared to deal with vulnerable situations (Barnard & Curry, 2011; Neff, 2016; Zhang et al., 2019).

This general conclusion is further supported by the results obtained for the different specific scenarios. For example, in Study 2, participants evaluated showing physical imperfections less positively for themselves than when others showed this potential vulnerability. These self–other differences, however, were attenuated for participants high in self-compassion. This finding is in line with the research demonstrating that self-compassion is associated with lower body shame and higher body appreciation (Albertson et al., 2015) as well as less perfectionism (Brown, 2012; Williams et al., 2008). Similarly, our findings reflect prior research demonstrating that self-compassion can help people to experience less defensiveness and distress when acknowledging their mistakes (Leary et al., 2007) or to rely less on avoidance and escape as coping strategies (Allen & Leary, 2010). In Study 3, we observed that individuals high (vs. low) in self-compassion exhibited reduced self–other differences in the evaluation of a mistake confession.

The observed results further contribute to a deeper conceptual understanding of self-compassion by testing its discriminant validity. Going beyond demonstrating the reliability of the moderation pattern, the obtained moderating effect of self-compassion remained stable even when controlling for other traits that are associated with self-compassion. Specifically, neither including self-esteem nor neuroticism eliminated the observed moderation. These results suggest that it is, indeed, self-compassion that allows people to see their display of vulnerabilities more positively rather than other traits that are associated with self-compassion. The present research, thus, contributes to the debate of the discriminant validity of self-compassion with respect to highly correlated traits such as self-esteem (Leary et al., 2007) or neuroticism (Pfattheicher et al., 2017).

Furthermore, in all studies, the hypothesized attenuation of self–other differences was driven by self-compassion improving the evaluation of actors. By contrast, only in Study 1a did we find a marginal trend toward more positive evaluations of other’s vulnerability display in self-compassionate individuals, whereas in the remaining three studies, this link remained nonsignificant. One possible explanation for the differences in the present set of studies rests on a crucial methodological difference: Self-compassion tended to influence the evaluation of others’ vulnerability display only when self-compassion was made accessible right before the evaluations. This activation of self-compassion might have strengthened the otherwise weak effect of self-compassion on evaluations of others, thus eliciting more pronounced differences.

Even though compassion and self-compassion are often seen as closely related constructs, prior research has predominantly investigated them separately (López et al., 2018), and the existing studies on the link between the two constructs yielded mixed results: Whereas some studies reported a positive link between self-compassion and compassion for others (Fuochi et al., 2018; Neff & Pommier, 2013), several studies failed to detect such a relationship (Barnard & Curry, 2011; Beresford, 2016; Leary et al., 2007; López et al., 2018) or even found a reverse correlation (Mills et al., 2018). The presented findings address the lack of data on this issue and contribute to the body of literature indicating that self-compassion may not necessarily impact how people feel for and about others.

### Future Research/Outlook

Notwithstanding the substantial evidence in support of our hypotheses, there are several questions that deserve additional attention. First, in all studies, self-compassion was measured as a stable trait. Although this conceptualization of self-compassion is in accordance with its origins (Neff, 2003), studies have shown that it is possible to influence self-compassion through interventions (e.g., Albertson et al., 2015). Therefore, future research might investigate whether the reduction of the BME can be achieved through directly teaching individuals to be more self-compassionate. In addition, although we tested the most likely candidates for alternative explanations—self-esteem and neuroticism—the impact of other related variables cannot be ruled out, creating further need for experimental designs in future research.

Second, although we provide evidence that self-compassion can help individuals to overcome the misperceptions about their own vulnerability display, we did not directly address the processes through which this effect occurs. Arguably, self-compassion might offer the documented benefits by equipping individuals with self-soothing tools to achieve quicker self-forgiveness and acceptance. Alternatively, self-compassion may reduce the BME by helping individuals experience less anxiety in the first place. However, if the second explanation were correct, we would have likely found at least some effect of neuroticism in Study 3. Yet, the general propensity to feel negative emotions neither moderated the BME, nor revealed a three-way interaction with self-compassion. These findings render it unlikely that self-compassion helps individuals in vulnerable situations by letting them bypass difficult emotions. Rather, it seems to lend a helping hand when dealing with the challenges of being vulnerable.

The latter possibility would also be in line with the underlying process that has been identified for the BME (Bruk et al., 2018): increased psychological distance that results in more abstract mental representations (= higher construal levels; Trope & Liberman, 2010) for others than the self. As our studies suggest that self-compassion may help individuals to see their own display of vulnerability in a more positive light, one intriguing implication of our findings, thus, may be that self-compassion may increase distance to the self through facilitating the perception of the self from the perspective of others. After all, self-compassion is, by definition, about treating oneself as a benevolent other, recognizing that others face similar challenges, and keeping a certain distance from one’s struggles through mindfulness. Therefore, the idea that all dimensions of self-compassion may influence construal level could be worth pursuing in future research.

The presented studies may also hold interesting implications for broader research on self–other differences in perception. Although in vulnerable situations, self-compassion helped to improve the overly negative perception of the self, in general, self-compassion does not necessarily lead to a more positive self-perception but rather to a less distorted and more grounded one (Neff, 2011). Therefore, although in many other areas of research on self–other differences, the self is elevated above others—the opposite of the BME pattern—self-compassion may still help to attenuate these self–other differences by creating a clearer lens on the self. For instance, it may decrease the self-serving attributions (Mezulis et al., 2004) by nudging people toward a more nuanced rather than just positive self-image. Similarly, self-compassion may curb the tendency toward unrealistic optimism around one’s own chances of success or failure (Weinstein, 1980), for instance, by reducing the illusion of control through a more mindful and realistic perception and by incorporating the notion that we all stumble. If pursued in future research, these considerations hold the potential for a more refined understanding of self–other differences beyond vulnerable situations.

Another important avenue for future research may center on the poorly understood link between self-compassion and compassion for others. Although our data did not reveal a consistent significant association, we did not test the null hypothesis of no effect nor would we argue that there is none. Rather, due to the inconsistency of previous research, we did not expect a substantial, robust association. Given that both positive (Fuochi et al., 2018) and negative (Mills et al., 2018) correlations between the two constructs have been observed before, such mixed results suggest nuance and complexity that warrant further investigations of potential moderators.

Notwithstanding these open questions, the reported findings provide substantial evidence for the crucial role of self-compassion in overcoming misperceptions about one’s own vulnerability displays. The vulnerable situations investigated in this manuscript are part of everyday life. Everyone will eventually make mistakes or be confronted with their imperfections. Extending kindness to oneself, reminding oneself that suffering is universal as well as holding one’s difficult experiences in mindful awareness seem to help people to see their vulnerabilities in a more positive light. In turn, a more positive outlook on showing vulnerability could open the door to all kinds of associated benefits, such as fulfilling personal relationships (Wheeless & Grotz, 1977), enhanced job performance and satisfaction (Brooks et al., 2015), or better health (Cepeda-Benito & Short, 1998).

## Supplemental Material

sj-docx-1-psp-10.1177_01461672211031080 – Supplemental material for You and I Both: Self-Compassion Reduces Self–Other Differences in Evaluation of Showing Vulnerability Click here for additional data file. Supplemental material, sj-docx-1-psp-10.1177_01461672211031080 for You and I Both: Self-Compassion Reduces Self–Other Differences in Evaluation of Showing Vulnerability by Anna Bruk, Sabine G. Scholl and Herbert Bless in Personality and Social Psychology Bulletin

sj-docx-2-psp-10.1177_01461672211031080 – Supplemental material for You and I Both: Self-Compassion Reduces Self–Other Differences in Evaluation of Showing Vulnerability Click here for additional data file. Supplemental material, sj-docx-2-psp-10.1177_01461672211031080 for You and I Both: Self-Compassion Reduces Self–Other Differences in Evaluation of Showing Vulnerability by Anna Bruk, Sabine G. Scholl and Herbert Bless in Personality and Social Psychology Bulletin

## Notes

1 Studies 1a to 3 asked participants whether they had followed the instructions while filling out the questionnaire, specifically whether they had differentiated between their own perspective and the perspective of another person or whether they had pictured both scenarios from the same perspective (either one’s own or another person’s). The majority of participants in all studies passed this manipulation check. Those who did not pass it largely stated that they imagined both situations from their own perspective. Presumably, this observation is due to the wording of the question as subsequent informal probing suggested that by saying that they imagined the other person from their own perspective, participants meant that they put themselves in the other person’s shoes. Importantly, in all four studies, we observed the same pattern of results (with comparable mean differences) when we excluded participants who had failed the manipulation check (because of the reduced *N*, these analyses, of course, have less power).

2 The effects of the control variable differences in vulnerability perception (see Supplemental Material) were inconsistent, suggesting that the variable does not play a crucial role in the observed findings. Importantly, the hypothesized interaction remained significant with or without the inclusion of this variable.

3 In Studies 1a, 1b, and 2, all main and interaction effects involving order were nonsignificant—a total of 16 effects. Surprisingly, in Study 3, three out of eight order effects were significant. Given that, overall, the pattern of results was independent of the emergence of order effects, we refrained from reporting these effects. We can, however, provide more detailed information upon request.

4 The results of exploratory post hoc gender analyses can be found in Supplemental Material.

5 Researchers from several laboratories contributed a total of 29 traits, attitudes, and behavioral measures to the online questionnaire. Only the trait self-compassion was included for the purpose of this study.

6 The traits measured in the online questionnaire in Study 1b included neuroticism, assessed with four items. The construct was contributed to the questionnaire by other researchers for an unrelated project. When new research (Pfattheicher et al., 2017) raised concerns about the potential overlap between self-compassion and neuroticism, we were able to include neuroticism in post hoc data analyses of Study 1b. The effects of neuroticism were nonsignificant and the inclusion of the construct did not change the previously observed pattern of results. However, given that the study did not have sufficient power for two simultaneous moderators, the measurement of neuroticism was relatively unreliable, and the findings were obtained in exploratory post hoc analyses, they can be interpreted only with great caution. Therefore, to rule out the possible effects of neuroticism, we conducted Study 3 to test an a priori hypothesis with sufficient power and reliability of measurement, and refrained from reporting the post hoc analyses from Study 1b. Detailed information, however, can be provided upon request.
