# Supplementary material for: You and I Both: Self-Compassion Reduces Self–Other Differences in Evaluation of Showing Vulnerability
Source: Pers Soc Psychol Bull. 2021 Jul 22;48(7):1054–67. doi: 10.1177/01461672211031080 (PMC9178778; doi:10.1177/01461672211031080)
Supplement: sj-docx-1-psp-10.1177_01461672211031080 – Supplemental material for You and I Both: Self-Compassion Reduces Self–Other Differences in Evaluation of Showing Vulnerability [file sj-docx-1-psp-10.1177_01461672211031080.docx]

**You and I both: Self-compassion reduces self–other differences in evaluation**

**of showing vulnerability**

Methodology File

Note: As all studies were conducted in Germany, the original material is in German. The present file includes the English translation of the material. The original German material can be requested from the first author.

The *oneself* and *others* conditions for female and male participants are combined in the following order: oneself_female_/oneself_male_ (others_female_ / others_male_)

Scenario for showing vulnerability by confessing love (Studies 1a and 1b):

(Bruk, Scholl, & Bless, 2018)

Please imagine the following situation.

You (Stephanie/Stephan) have (has) a very good friend with whom you (she/he) spend(s) lots of time and you (they) get along perfectly. You (They) have many interests in common and enjoy talking to each other, both about everyday things and about life’s bigger questions. He/She supports you (Stephanie/Stephan) with important decisions and in difficult situations.

After a while you (Stephanie/Stephan) noticed that you (she/he) have (has) gradually developed deeper feelings for your (her/his) good friend. You (She/He) are (is) starting to realize that you (she/he) are (is) deeply in love. You (She/He) feel(s) really alive and in better mood when your (her/his) friend is around and even your (her/his) everyday life seems to be more exciting than usually. You (She/He) are (is) becoming more and more aware of the fact that you (she/he) wish(es) your (their) friendship would turn into a romantic relationship.

You (She/He) are (is) wondering whether you (she/he) should confess your (her/his) love to him/her. If he/she feels the same way about you (her/him), your (her/his) biggest dream will come true. Just thinking about how he/she puts his/her arms around you (her/him) and tells you (her/him) that he/she loves you (her/him) too makes you (her/him) grin from ear to ear. On the other hand, you (she/he) might lose one of your (her/his) best friends, if he/she has no interest in you (her/him). Besides, it would be a very unpleasant or even embarrassing situation if his/her reaction to your (her/his) confession were not positive. You (She/He) keep(s) picturing over and over again in your (her/his) mind how you (she/he) say(s) the words “I love you” but get(s) only silence in return. You (She/He) can already imagine sinking into the earth out of shame. Furthermore, if he/she had the same feelings for you (her/him), he/she could bring it up himself/herself, couldn’t he/she? You (She/He) are (is) wondering why you (she/he) should be the one to confess your (her/his) love first.

After turning the situation over in your (her/his) mind for a long time, you (she/he) confess(es) your (her/his) love to him/her. You (Stephanie/Stephan) feel(s) vulnerable and are (is) waiting for a reaction.

Perception of vulnerability for confessing love (Studies 1a and 1b):

(Bruk, Scholl, & Bless, 2018)

Scale:1 (*strongly disagree*) to 7 (*strongly agree*)

Please indicate to what extent you agree with the following statement:

- By confessing love first, I am (Stephanie/Stephan is) showing my (her/his) vulnerability.

Evaluation of showing vulnerability (Study 1a and Study 1b):

(Bruk, Scholl, & Bless, 2018)

Scale:1 (*strongly disagree*) to 7 (*strongly agree*)

(R= reversed item)

Please indicate to what extent you agree with the following statements:

- By showing my (her/his) vulnerability, I am (Stephanie/Stephan is) showing weakness. (R)
- By showing my (her/his) vulnerability, I am (Stephanie/Stephan is) showing courage.
- By showing my (her/his) vulnerability, I am (Stephanie/Stephan is) showing my (her/his) inadequacy. (R)
- By showing my (her/his) vulnerability, I am (Stephanie/Stephan is) showing strength.
- Generally, when I (Stephanie/ Stephan) show(s) my (her/his) vulnerability, other people find it repellent. (R)
- Generally, I (Stephanie/ Stephan) should avoid showing my (her/his) vulnerability. (R)
- Generally, it is good for me (Stephanie/ Stephan) to show my (her/his) vulnerability.
- Generally, when I (Stephanie/ Stephan) show(s) my (her/his) vulnerability, other people find it desirable.

Scenario for showing vulnerability by revealing physical imperfections (Study 2):

(Bruk, Scholl, & Bless, 2018)

Please imagine the following situation.

It’s the first hot summer day. Your (Christiane’s/ Christian’s) best friend calls you (her/him) and asks if you (Christiane/Christian) want(s) to go to the swimming pool with the clique. That would be just perfect given the current weather! A guy/girl on whom you’ve (Christiane/ Christian has) had a crush for months will be there, too. Sounds like a great afternoon. However, you are (she/he is) still hesitant to respond.

You (She/He) take(s) a critical look in the mirror. You do (Christiane/Christian does) work out and your (her/his) nutrition is not that unhealthy either but your (her/his) winter fat seems to be resistant to that. On the other hand, you (she/he) really enjoy(s) swimming and being in the sun. In addition, your (her/his) friends would be glad if you (she/he) came along and it would be a fun day for sure. Nevertheless, you (she/he) would feel really uncomfortable if you (she/he) got contemptuous looks from the others. Your (Christiane’s/Christian’s) friends have always been more athletic than you (she/he) and one can tell that just by looking at them. Plus, the girls/guys from your (her/his) clique don’t exactly mince matters and could make a stupid joke about your (her/his) winter fat. Your (Her/His) friends just don’t know what it’s like to be dissatisfied with your own body and therefore don’t understand how hurtful such flippant remarks can be. Just the mere thought of such a remark makes you (her/him) sink into the earth out of shame. Especially, if your (her/his) crush is there too, you (she/he) would like to impress him/her a little. But what if he/she doesn’t find your (her/his) body attractive? Maybe you (Christiane/Christian) should just stay at home and wait until you (she/he) are (is) in better shape?

After turning the situation over in your (her/ his) mind for a long time, you (she/he) decide(s) to go to the swimming pool. You (She/He) feel(s) vulnerable and are (is) waiting for the reactions.

Perception of vulnerability for revealing physical imperfections (Study 2):

(Bruk, Scholl, & Bless, 2018)

Scale:1 (*strongly disagree*) to 7 (*strongly agree*)

Please indicate to what extent you agree with the following statement:

- By revealing imperfections of my (her/his) body, I am (Christiane/Christian is) showing my (her/his) vulnerability.

Evaluation of showing vulnerability (Study 2):

(Bruk, Scholl, & Bless, 2018)

Scale:1 (*strongly disagree*) to 7 (*strongly agree*)

(R= reversed item)

Please indicate to what extent you agree with the following statements:

- By showing my (her/his) vulnerability, I am (Christiane/Christian is) showing weakness. (R)
- By showing my (her/his) vulnerability, I am (Christiane/Christian is) showing courage.
- By showing my (her/his) vulnerability, I am (Christiane/Christian is) showing my (her/his) inadequacy. (R)
- By showing my (her/his) vulnerability, I am (Christiane/Christian is) showing strength.
- Generally, when I (Christiane/Christian) show(s) my (her/his) vulnerability, other people find it repellent. (R)
- Generally, I (Christiane/Christian) should avoid showing my (her/his) vulnerability. (R)
- Generally, it is good for me (Christiane/Christian) to show my (her/his) vulnerability.
- Generally, when I (Christiane/Christian) show(s) my (her/his) vulnerability, other people find it desirable.

Scenario for showing vulnerability by admitting a mistake (Study 3):

(Bruk, Scholl, & Bless, 2018)

Please imagine the following situation.

You (Michaela/Michael) work(s) for a company with more than 50 employees. You (She/He) very much enjoy(s) working for this company. There you’ve (she’s/he’s) got a lot of autonomy, nice colleagues, and a good salary. Currently you (she/he) are (is) working on a big project that is of high importance to your (her/his) employer because a vast amount of money has been invested in it, and the company’s image partly depends on the success of this particular project.

However, you (Michaela/Michael) have (has) recently noticed having made a mistake in your (her/his) work. You (She/He) are (is) not sure whether the mistake could have serious consequences in later stages of the project or if it might not impact the project at all. One thing is for sure: The mistake cannot be traced back to you (her/him). So you (she/he) are (is) wondering: Should you (she/he) admit the mistake?

You (She/He) are (is) genuinely sorry that your (her/his) mistake caused more work for your (her/his) colleagues and might have impaired the project’s success. By admitting your (her/his) mistake you (Michaela/Michael) could at least make amends, and you (she/he) imagine(s) it to be a great relief. Nevertheless, you (he/she) don’t (doesn’t) really know how your (her/his) boss would react to such a confession. Maybe she/he would realize that although you (Michaela/Michael) have (has) made a mistake, it is very honest of you (her/him) to admit it and would have more trust in you (her/him). On the other hand, there is a possibility that she/he might only focus on the mistake and might lose her/his trust in your (Michaela’s/Michael’s) professional expertise. And what if the incident also influences your (Michaela’s/Michael’s) chances of promotion? Would you (she/he) be assigned other, less important tasks that would likely bore you (her/him) and not challenge you (her/him) at all? Besides, you (she/he) feel(s) really embarrassed that you (she/he) made such a stupid mistake. You (She/He) should have known better. The mere thought of telling your (her/his) boss about it makes you (her/him) sink into the earth out of shame.

After turning the situation over in your (her/his) mind for a long time, you (she/he) admit(s) your (her/his) mistake. You (She/He) feel(s) vulnerable and are (is) waiting for a reaction.

Perception of vulnerability for admitting a mistake (Study 3):

(Bruk, Scholl, & Bless, 2018)

Scale:1 (*strongly disagree*) to 7 (*strongly agree*)

Please indicate to what extent you agree with the following statement:

- By admitting my (her/his) mistake, I am (Michaela/Michael is) showing my (her/his) vulnerability.

Evaluation of showing vulnerability (Study 3):

(Bruk, Scholl, & Bless, 2018)

Scale:1 (*strongly disagree*) to 7 (*strongly agree*)

(R= reversed item)

Please indicate to what extent you agree with the following statements:

- By showing my (her/his) vulnerability, I am (Michaela/Michael is) showing weakness. (R)
- By showing my (her/his) vulnerability, I am (Michaela/Michael is) showing courage.
- By showing my (her/his) vulnerability, I am (Michaela/Michael is) showing my (her/his) inadequacy. (R)
- By showing my (her/his) vulnerability, I am (Michaela/Michael is) showing strength.
- Generally, when I (Michaela/Michael) show(s) my (her/his) vulnerability, other people find it repellent. (R)
- Generally, I (Michaela/Michael) should avoid showing my (her/his) vulnerability. (R)
- Generally, it is good for me (Michaela/Michael) to show my (her/his) vulnerability.
- Generally, when I (Michaela/Michael) show(s) my (her/his) vulnerability, other people find it desirable.

Instruction for the trait items (Study 1a to Study 3):

You will now read a series of statements, each describing certain (general) human characteristics or reactions, all of which have something to do with feelings. Please state to what extent these statements apply to you. Maybe you can think of a concrete experience for one or the other general description. There are no right or wrong answers.

We thank you for your cooperation and your honest response to the questions. Please start now by clicking on "continue".

Self-compassion scale (Study 1a to Study 3):

(Dyllick-Brenzinger, 2010; Neff, 2003)

Scale:1 (*strongly disagree*) to 7 (*strongly agree*)

(SK= Self-Kindness Subscale; SJ = Self-Judgment Subscale; CH = Common Humanity Subscale; I = Isolation Subscale; M = Mindfulness Subscale; O = Over-Identification Subscale)

- I’m disapproving and judgmental about my own flaws and inadequacies. (SJ)
- When I’m feeling down I tend to obsess and fixate on everything that’s wrong. (O)
- When things are going badly for me, I see the difficulties as part of life that everyone goes through. (CH)
- When I think about my inadequacies it tends to make me feel more separate and cut off from the rest of the world. (I)
- I try to be loving towards myself when I’m feeling emotional pain. (SK)
- When I fail at something important to me I become consumed by feelings of inadequacy. (O)
- When I’m down and out, I remind myself that there are lots of other people in the world feeling like I am. (CH)
- When times are really difficult, I tend to be tough on myself. (SJ)
- When something upsets me I try to keep my emotions in balance. (M)
- When I feel inadequate in some way, I try to remind myself that feelings of inadequacy are shared by most people. (CH)
- I’m intolerant and impatient towards those aspects of my personality I don’t like. (SJ)
- When I’m going through a very hard time, I give myself the caring and tenderness I need. (SK)
- When I’m feeling down I tend to feel like most other people are probably happier than I am. (I)
- When something painful happens I try to take a balanced view of the situation. (M)
- I try to see my failings as part of the human condition. (CH)
- When I see aspects of myself that I don’t like, I get down on myself. (SJ)
- When I fail at something important to me I try to keep things in perspective. (M)
- When I’m really struggling I tend to feel like other people must be having an easier time of it. (I)
- I’m kind to myself when I’m experiencing suffering. (SK)
- When something upsets me I get carried away with my feelings. (O)
- I can be a bit cold-hearted towards myself when I’m experiencing suffering. (SJ)
- When I’m feeling down I try to approach my feelings with curiosity and openness. (M)
- I’m tolerant of my own flaws and inadequacies. (SK)
- When something painful happens I tend to blow the incident out of proportion. (O)
- When I fail at something that’s important to me I tend to feel alone in my failure. (I)
- I try to be understanding and patient towards those aspects of my personality I don’t like. (SK)

Self-esteem scale (Study 2):

(Ferring & Filipp, 1996; Rosenberg, 1965)

Scale: 1 (*strongly disagree*) to 7 (*strongly agree*)

(R= reversed item)

- On the whole, I am satisfied with myself.
- At times I think I am no good at all. (R)
- I feel that I have a number of good qualities.
- I am able to do things as well as most other people.
- I feel I do not have much to be proud of. (R)
- I certainly feel useless at times. (R)
- I feel that I'm a person of worth.
- I wish I could have more respect for myself. (R)
- All in all, I am inclined to think that I am a failure. (R)
- I take a positive attitude toward myself.

Neuroticism scale (Study 3):

(Borkenau & Ostendorf, 2008; Costa & McCrea, 1992)

Scale: 1 = *strongly disagree*; 2 = *disagree*; 3 = *neutral*; 4 = *agree*; 5 = *strongly agree*

(R= reversed item)

- I’m not a worrier. (R)
- I often feel inferior to others.
- When I’m under a great deal of stress, sometimes I feel like I’m going to pieces.
- I rarely feel lonely or blue. (R)
- I often feel tense and jittery.
- Sometimes I feel completely worthless.
- I rarely feel fearful or anxious. (R)
- I often get angry at the way people treat me.
- Too often, when things go wrong, I get discouraged and feel like giving up.
- I am seldom sad or depressed. (R)
- I often feel helpless and want someone else to solve my problems.
- At times I have been so ashamed I just wanted to hide.

References:

Borkenau, P., & Ostendorf, F. (2008). *NEO-Fünf-Faktoren Inventar: nach Costa u. McCrae; NEO-FFI*. Göttingen: Hogrefe.

Bruk, A., Scholl, S. G., & Bless, H. (2018). Beautiful mess effect: Self–other differences in evaluation of showing vulnerability. *Journal of Personality and Social Psychology, 115*(2), 192–205.

Costa, P. T., & McCrea, R. R. (1992). *Revised Neo Personality Inventory (NEO Pi-R) and Neo Five-Factor Inventory (NEO-FFI)*. Psychological Assessment Resources.

Dyllick-Brenzinger, T. (2010). *Self-Compassion: Zusammenhang mit Schuld, Scham und erinnertem Erziehungsverhalten*. Mannheim: Unveröffentlichte Diplomarbeit.

Ferring, D. & Filipp, S.-H. (1996). Messung des Selbstwertgefühls: Befunde zu Reliabilität, Validität und Stabilität der Rosenberg-Skala. *Diagnostica-Gottingen*, *42*, 284–292.

Neff, K. D. (2003). The development and validation of a scale to self-compassion. *Self and Identity, 2*(3), 223–250.

Rosenberg, M. (1965). *Society and the adolescent self-image*. Princeton: Princeton University Press.
