# Supplementary material for: You and I Both: Self-Compassion Reduces Self–Other Differences in Evaluation of Showing Vulnerability
Source: Pers Soc Psychol Bull. 2021 Jul 22;48(7):1054–67. doi: 10.1177/01461672211031080 (PMC9178778; doi:10.1177/01461672211031080)
Supplement: sj-docx-2-psp-10.1177_01461672211031080 – Supplemental material for You and I Both: Self-Compassion Reduces Self–Other Differences in Evaluation of Showing Vulnerability [file sj-docx-2-psp-10.1177_01461672211031080.docx]

**You and I both: Self-compassion reduces self–other differences in evaluation**

**of showing vulnerability**

Supplementary Analyses

**Effects of Differences in Vulnerability Perception (DVP)**

**Study 1a:**

DVP: *F*(1, 55) = 3.18, *p =* .08, η_p_^2^ = .06

DVP * role: *F*(1, 55) = 16.53, *p <* .001, η_p_^2^ = .23 (When participants perceived the described situation as more vulnerable for the self than others, the self-other differences in the evaluations of showing vulnerability increased.)

**Study 1b:**

DVP: *F*<1

DVP * role: *F*<1

**Study 2:**

DVP: *F*(1, 88) = 2.02, *p =* .16, η_p_^2^ = .02

DVP * role: *F*(1, 88) = 21.78, *p <* .001, η_p_^2^ = .20 (When participants perceived the described situation as more vulnerable for the self than others, the self-other differences in the evaluations of showing vulnerability increased.)

**Study 3:**

DVP: *F*<1

DVP * role: *F*(1, 92) = 1.46, *p =* .23, η_p_^2^ = .02

**Effects of Gender**

**Study 1a:**

Gender*: F*<1

Gender * role: *F*(1, 54) = 1.79, *p =* .19, η_p_^2^ = .03

**Study 1b:**

Gender*: F*<1

Gender * role: *F*(1, 76) = 7.85, *p =* .01, η_p_^2^ = .09 (The self–other differences in the evaluation of showing vulnerability were more pronounced for women than men)

**Study 2:**

Gender*: F*<1

Gender * role: *F*(1, 88) = 2.38, *p =* .13, η_p_^2^ = .03

**Study 3:**

Gender*: F*(1, 91) = 5.77, *p =* .02, η_p_^2^ = .06 (Showing vulnerability was evaluated more positively by women than men)

Gender * role: *F*(1, 91) = 2.57, *p =* .11, η_p_^2^ = .03

Importantly, controlling for gender did not change the pattern of the hypothesized results: The effects of role as well the role by self-compassion interaction remained significant in all four studies, whereas the role by self-esteem interaction (Study 2) and the role by neuroticism interaction (Study 3) remained non-significant.
